# Impact of an Embedded Palliative Care Clinic on Healthcare Utilization for Patients With a New Thoracic Malignancy

**DOI:** 10.3389/fonc.2022.835881

**Published:** 2022-02-28

**Authors:** Kelly C. Gast, Jason A. Benedict, Madison Grogan, Sarah Janse, Maureen Saphire, Pooja Kumar, Erin M. Bertino, Julia L. Agne, Carolyn J. Presley

**Affiliations:** ^1^ Division of Medical Oncology, The Ohio State University James Comprehensive Cancer Center, Columbus, OH, United States; ^2^ Center for Biostatistics, The Ohio State University Wexner Medical Center, Columbus, OH, United States; ^3^ Department of Pharmacy, The Ohio State University James Comprehensive Cancer Center, Columbus, OH, United States; ^4^ Division of Palliative Medicine, The Ohio State University Wexner Medical Center, Columbus, OH, United States

**Keywords:** thoracic, healthcare utilization, embedded, palliative care, lung cancer

## Abstract

**Introduction:**

Palliative care is beneficial for patients with advanced lung cancer, but the optimal model of palliative care delivery is unknown. We investigated healthcare utilization before and after embedding a palliative care physician within a thoracic medical oncology “onco-pall” clinic.

**Methods:**

This is a retrospective cross-sectional cohort study comparing healthcare outcomes in two cohorts: “pre-cohort” 12 months prior to and “post-cohort” 12-months after the onco-pall clinic start date. Patients were included if they had a new diagnosis of lung cancer and received care at The Ohio State University Thoracic Oncology Center, and resided in Franklin County or 6 adjacent counties. During the pre-cohort time period, access to palliative care was available at a stand-alone palliative care clinic. Palliative care intervention in both cohorts included symptom assessment and management, advance care planning, and goals of care discussion as appropriate. Outcomes evaluated included rates of emergency department (ED) visits, hospital admissions, 30-day readmissions, and intensive care unit (ICU) admissions. Estimates were calculated in rates per-person-years and with Poisson regression models.

**Results:**

In total, 474 patients met criteria for analysis (214 patients included in the pre-cohort and 260 patients in the post-cohort). Among all patients, 52% were male and 48% were female with a median age of 65 years (range 31-92). Most patients had non-small cell lung cancer (NSCLC - 17% stage 1-2, 20% stage 3, 47% stage 4) and 16% had small cell lung cancer. The post-cohort was older [median age 66 years vs 63 years in the pre-cohort (p-value: < 0.01)]. The post-cohort had a 26% reduction in ED visits compared to the pre-cohort, controlling for age, race, marital status, sex, county, Charlson score at baseline, cancer type and stage (adjusted relative risk: aRR: 0.74, 95% CI: 0.58-0.94, p-value = 0.01). Although not statistically significant, there was a 29% decrease in ICU admissions (aRR: 0.71, 95% CI: 0.41-1.21, p-value = 0.21) and a 15% decrease in hospital admissions (aRR: 0.85, 95% CI: 0.70-1.03, p-value = 0.10). There was no difference in 30-day readmissions (aRR: 1.03, 95% CI: 0.73-1.45, p-value = 0.85).

**Conclusions:**

Embedding palliative care clinics within medical oncology clinics may decrease healthcare utilization for patients with thoracic malignancies. Further evaluation of this model is warranted.

## Introduction

Multiple randomized trials have demonstrated that patients with advanced lung cancer benefit from early palliative care (within 8 weeks of diagnosis) concurrently with standard oncology care (1–4). These benefits include improved health-related quality of life (1–3, 5) and survival (1, 6). As a result, the American Society of Clinical Oncology guidelines recommend all patients with advanced cancer receive early interdisciplinary palliative care services (7). Despite the known benefits to early palliative care, there have been challenges in implementing this recommendation. These challenges include resource availability, financial constraints, increased clinic appointments, patient and caregiver fatigue, and patient and provider perceptions of end-of-life care (8, 9).

Although access to palliative care has increased in recent years, the optimal model of palliative care delivery is unknown. There has been a significant increase in outpatient palliative care clinics at National Cancer Institute (NCI)-designated cancer centers (95% in 2018 compared to 59% in 2009) (10). However, the vast majority of NCI-designated cancer centers offer stand-alone outpatient clinics (82.7%) while a smaller proportion offer palliative care in oncology clinics (38.5%) (10).

Several studies have evaluated the feasibility and impact of this newer model of embedded palliative care delivery (11–14). Embedded care models increased palliative care referral rates, resulted in palliative care intervention earlier in the disease course, and improved symptom burden (11, 12). While embedded palliative care in oncology clinics improves access, reported results on the effect on healthcare outcomes is mixed. Multiple studies have reported that the embedded palliative care model improves advanced care planning discussions (13, 14). One study showed increased hospice referral rates (13), while two studies demonstrated no difference (12, 14). There was no difference reported between embedded and stand-alone palliative clinic models with respect to end-of-life quality metrics including hospital or intensive care unit (ICU) admissions in the last 30 days of life (13, 14), emergency department (ED) visits (14), or receipt of chemotherapy in the last 14 days of life (12, 14). In addition to improving the accessibility of palliative care, there has been increased focus on improving the quality of palliative care services (15). A few studies have evaluated the effect of early vs delayed palliative care on improving healthcare outcomes prior to death. However, these studies found no difference in hospitalizations, ICU admissions, ED visits, or chemotherapy in the last 14 days of life (6, 16).

As embedded palliative care clinics become more prevalent within the oncology field, more research is necessary to demonstrate what effect this model may have on healthcare utilization outcomes. To address this knowledge gap, we evaluated the effect of the onco-pall embedded care model on healthcare utilization outcomes across the course of the disease rather than limited to the immediate days preceding end-of-life. In this study, we investigated the effect of a new onco-pall embedded care model available to providers within one Thoracic Oncology clinic at a large NCI-designated cancer center on healthcare utilization outcomes compared to a stand-alone palliative care clinic model. We hypothesized that an embedded onco-palliative care model would decrease ED visits, hospital admissions, ICU admissions, and 30-day readmissions compared to a stand-alone palliative care clinic model.

## Materials and Methods

### Study Design

This was a retrospective study of patients with a diagnosis of thoracic malignancy seen by Thoracic Medical Oncology at The Ohio State University both before and after the establishment of a new embedded palliative care provider within the Thoracic Oncology clinic. Prior to development of this embedded onco-pall clinic, outpatient palliative care was only available *via* stand-alone clinic model that operates independently and in a separate location from oncology clinics. The stand-alone palliative clinic has its own dedicated interdisciplinary team including nursing staff, social work, chaplain, and clinical pharmacist. The embedded palliative care clinic model included one palliative care physician who shared clinical workspace with the medical oncology team two full days per week. The palliative care physician was flexible in scheduling and was available to evaluate the patient at the most convenient time within the clinic flow (before, after, or with the medical oncologist, or during the infusion visit). Oncology clinical resources, including nursing staff, scheduling assistants, case managers, social workers, and pharmacists, were shared with the embedded palliative physician. In both clinic models, palliative clinic referrals are ordered per discretion of the oncology team without triggering referral guidelines. Patients seen in both the stand-alone and embedded clinic models received the same content of palliative care intervention including symptom assessment and management, advance care planning, and goals of care discussion as appropriate. End of life discussions in the embedded clinic model differed in that oncology and palliative providers frequently shared these encounters with the patient and family. All palliative clinic patients are seen monthly until symptom management has stabilized and then every 6-8 weeks thereafter unless sooner follow up is requested by the patient or oncology team. The Ohio State University Institutional Review Board approved this retrospective cross-sectional cohort study.

### Patient Population

Patients included in this retrospective cohort were ≥ 18 years of age with a diagnosis of lung cancer and visited the Thoracic Oncology clinic in the designated time frame per cohort. Patients were excluded from the study if they had visited the Thoracic Oncology clinic in the preceding 1 year prior to the respective study period. This allowed for only new patients to be included in each respective study period. Patients were required to be established patients with at least two visits during the study period. Patients were required to reside within the same county (Franklin County) or one of six counties adjacent to the academic medical center (hospital catchment area). Patients may have received systemic treatment, surgery, and radiation. Systemic treatment included chemotherapy, immunotherapy, and targeted therapy. Patients evaluated between September 1, 2017 and August 31, 2018 in the Thoracic Oncology clinic were included in the pre-cohort. During this time, palliative care was available by referral to a free-standing outpatient clinic located approximately 2 miles away from the Thoracic Oncology clinic. The embedded clinic opened on September 1, 2018 in a limited capacity. There was a five-month ramp-up period until the embedded palliative care clinic was functioning at full capacity. Therefore, patients were included in the post-cohort if they were evaluated in the Thoracic Oncology clinic between February 1, 2019 and January 31, 2020.

### Data Collection

Data was abstracted from the electronic medical record and included baseline demographics including age, gender, race/ethnicity, marital status, zip code, and county of residence. Clinical variables included cancer diagnosis at baseline, cancer stage at baseline, cancer treatment, Charlson comorbidity index at baseline, ED visits, hospital admissions, ICU admissions, 30-day readmissions, palliative care referral orders, and palliative care referral completion (defined as patient having been evaluated by palliative care at least once).

### Statistical Analyses

Descriptive statistics were used for baseline demographics and clinical variables. Categorical variables were summarized by counts and proportions and Fisher’s exact test was used for comparisons between the two cohorts. Continuous variables were summarized by median and range and comparisons between the cohorts were made using the Wilcoxon rank-sum test. Healthcare utilization outcomes, including estimates for ED visits, hospital admissions, ICU admissions, and 30-day readmissions, were calculated in rates-per-person years. Poisson regression models with robust sandwich-type standard errors and time as an offset were used for statistical analyses (17).

Patients were considered at risk for ED visits, hospital admissions, and ICU admissions following their first visit to the Thoracic Oncology clinic. For the outcome of 30-day readmissions, patients became at risk for the first 30 days following the discharge date of each hospital admission that was identified as an emergency or urgent admission unless a discharge occurred for the following reasons: 1) left against medical advice or discontinued care; 2) discharged/transferred to a designated cancer center; and 3) discharged/transferred to a short-term general hospital for inpatient care. If a patient returned within 30 days of their previous hospital discharge due to an elective procedure, this visit was not counted as a 30-day readmission. For each outcome of interest, patients exited the study when there was loss of follow-up, the patient died, or the study period ended for their cohort.

Models were adjusted for potential confounding by patient age, race (non-Hispanic white vs other), marital status (married vs unmarried), sex (male vs female), location (patient primary address within Franklin County vs adjacent county), Charlson score at baseline, and cancer type and stage at baseline (NSCLC stage 1 or 2, NSCLC stage 3, NSCLC stage 4, or SCLC). All confidence intervals are two-sided and presented at their nominal level. P-values < 0.05 were considered statistically significant. All analyses were performed in R version 4.0.0.

## Results

### Study Sample

**Figure 1:**
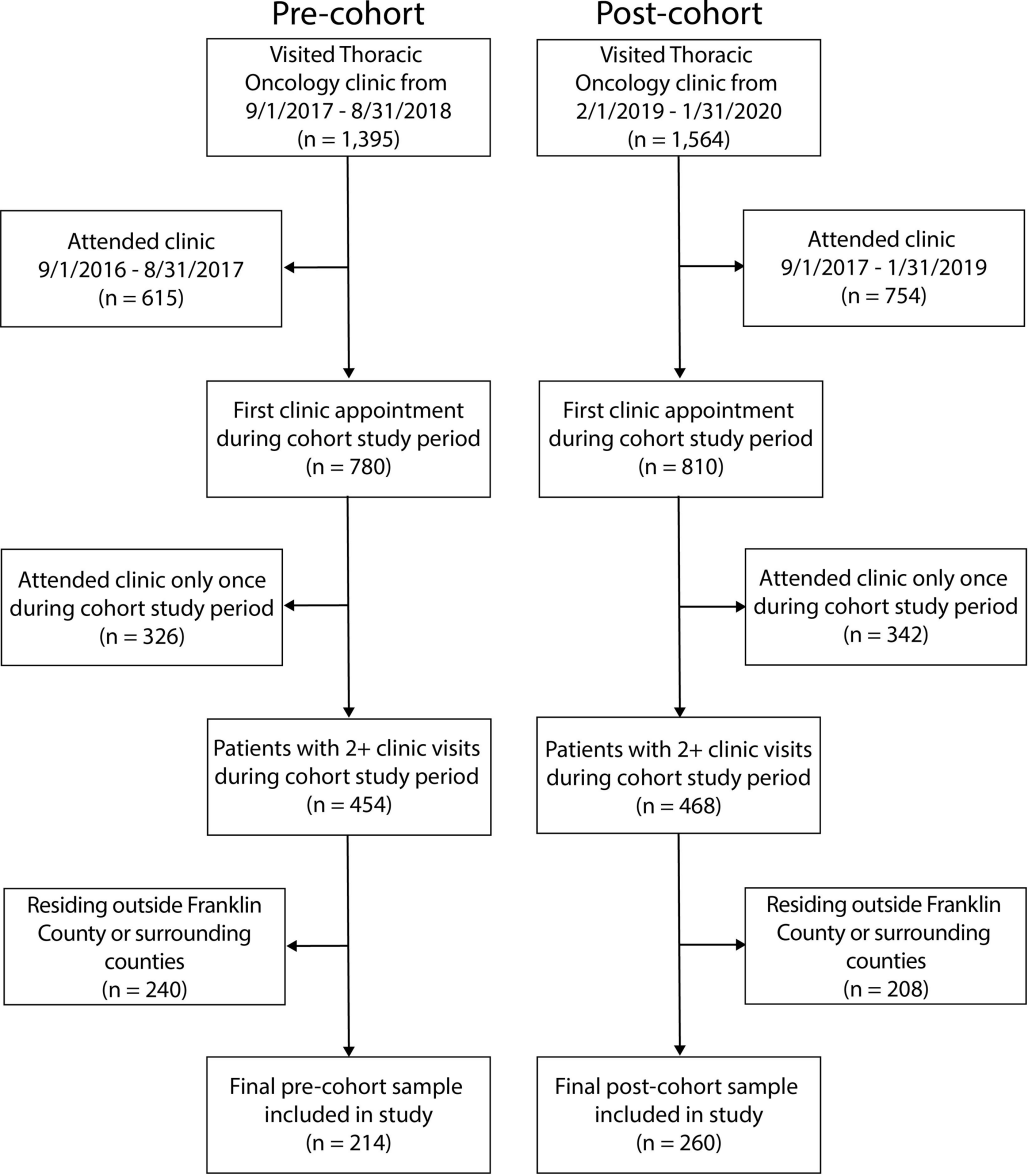
Cohort development.

The final analytical sample included 474 patients, 214 patients in the pre-cohort and 260 patients in the post-cohort **(** **Figure 1** **).** Among all patients, 52% were male and 48% were female with a median age of 65 years (y) (range 31-92 y). The majority of patients were non-Hispanic white (80%). The majority of patients (66%) resided within the same county as the academic medical center and 34% resided within a county adjacent to the academic medical center (hospital catchment area). Cancer type and stage included 17% NSCLC stage 1 or 2, 20% NSCLC stage 3, 47% NSCLC stage 4, and 16% SCLC. Treatment types included surgery (3%), radiation (26%), chemotherapy (35%), immunotherapy (20%), and other (3%). Patients in the post-cohort tended to be older and to have a higher Charlson comorbidity score. Additional details on patient characteristics, tumor characteristics, and treatment characteristics are available in **Table 1**.

**Table 1:** Patient characteristics.

Characteristic^1^	Overall (n = 474)	Pre-cohort (n = 214)	Post-cohort (n = 260)	P-value^3^
**Age,** median (min-max)	65 (31-92)	63 (31-86)	66 (37-92)	<0.01
**Age**				0.06
* Under 65*	228 (48.1)	116 (54.0)	112 (43.1)	
* 65+ and under 70*	87 (18.4)	33 (15.4)	54 (20.8)	
* 70+ and under 75*	77 (16.2)	37 (17.3)	40 (15.4)	
* 75+ and under 80*	59 (12.4)	21 (9.8)	38 (14.6)	
* 80+*	23 (4.9)	7 (3.3)	16 (6.2)	
**Sex**				>0.95
* Female*	227 (47.9)	102 (47.7)	125 (48.1)	
* Male*	247 (52.1)	112 (52.3)	135 (51.9)	
**Race/ethnicity**				0.82
* Non-Hispanic white*	377 (80.0)	172 (80.8)	205 (79.5)	
* Other*	94 (20)	41 (19.2)	53 (20.5)	
* Unknown*	3	1	2	
**Marital status**				0.41
* Unmarried*	229 (48.3)	108 (50.5)	121 (46.5)	
* Married*	245 (51.7)	106 (49.5)	139 (53.5)	
**Residing location**				0.85
* Within Franklin County*	312 (65.8)	142 (66.4)	170 (65.4)	
* Surrounding Franklin County*	162 (34.2)	72 (33.6)	90 (34.6)	
**Charlson comorbidity at baseline,** median (min-max)	6 (0-24)	5 (1-14)	6 (0-24)	0.07
**Cancer type and stage at baseline**				0.53
* NSCLC Stage 1 or 2*	82 (17.3)	40 (18.8)	42 (16.2)	
* NSCLC Stage 3*	94 (19.9)	40 (18.8)	54 (20.7)	
* NSCLC Stage 4*	222 (46.9)	104 (48.8)	118 (45.4)	
* SCLC*	75 (15.9)	29 (13.6)	46 (17.7)	
* Unknown*	1	1	0	
**Treatment undergone during study** ^2^				
* Chemotherapy*	165 (34.8)	84 (39.3)	81 (31.2)	0.07
* Radiation*	125 (26.4)	61 (28.5)	64 (24.6)	0.35
* Immunotherapy*	95 (20.0)	42 (19.6)	53 (20.4)	0.91
* Surgery*	15 (3.2)	10 (4.7)	5 (1.9)	0.11
* Other therapy*	15 (3.2)	12 (5.6)	3 (1.2)	0.01

^1^Presented as n (%) unless otherwise indicated.

^2^Patients could undergo more than one treatment during the course of the study.

^3^P-values calculated using Fisher’s exact test for categorical variables and Wilcoxon rank-sum test for continuous variables.

NSCLC, non-small cell lung cancer.

SCLC, small-cell lung cancer.

### Healthcare Utilization Outcomes

**Table 2** provides healthcare utilization outcomes for ED visits, hospital admissions, ICU admissions, and 30-day readmissions. Events per-person-year were decreased in the post-cohort relative to the pre-cohort for ED visits (2.54 vs. 3.43), hospital admissions (3.77 vs. 4.39) and ICU admissions (0.31 vs 0.46). There was no decrease in events per-person year for 30-day readmissions observed in the post-cohort (6.71) relative to the pre-cohort (6.58). There was a statistically significant 26% reduction in ED visits in the post-cohort relative to the pre-cohort (adjusted relative risk: 0.74, 95% CI: 0.58-0.94, p-value = 0.01). Although not statistically significant, we also observed a 29% reduction in ICU admissions (adjusted relative risk: 0.71, 95% CI: 0.41-1.21, p-value = 0.21) and a 15% reduction in hospital admissions (adjusted relative risk: 0.85, 95% CI: 0.70-1.03, p-value = 0.10) in the post-cohort relative to the pre-cohort. As this study was performed at a single site, it is likely that patients who live further from the site would be less likely to be captured in the analytic data as they may preferentially visit hospitals closer to their residences. Therefore, we performed a sensitivity analysis by re-analyzing all healthcare outcomes for patients residing only within Franklin County. This analysis yielded similar results **(Appendix Table 2)**.

**Table 2:** Healthcare utilization comparing pre- versus post-cohort.

	Number of events	Total person-years of exposure	Events per-person-year (95% CI)	Relative risk (95% CI)	Adjusted relative risk (95% CI)^1^
**ICU admissions**					
*Pre-cohort*	36	78.8	0.46 (0.32-0.63)	Reference	Reference
*Post-cohort*	30	96.4	0.31 (0.21-0.44)	0.68 (0.40-1.16)	0.71 (0.41-1.21)
**ED visits**					
*Pre-cohort*	270	78.8	3.43 (3.03-3.86)	Reference	Reference
*Post-cohort*	245	96.4	2.54 (2.23-2.88)	0.74 (0.58-0.94)	0.74 (0.58-0.94)
**Hospital Admissions**					
*Pre-cohort*	346	78.8	4.39 (3.94-4.88)	Reference	Reference
*Post-cohort*	363	96.4	3.77 (3.39-4.17)	0.86 (0.71-1.03)	0.85 (0.70-1.03)
**30-day readmissions^2^**					
*Pre-cohort*	74	11.2	6.58 (5.17-8.26)	Reference	Reference
*Post-cohort*	69	10.3	6.71 (5.22-8.49)	1.02 (0.71-1.46)	1.03 (0.73-1.45)

^1^Adjusted for age, race (Non-Hispanic white vs other), marital status (married vs unmarried), sex (male vs female), location (patient primary address within Franklin County vs adjacent county), Charlson score at baseline, cancer type and cancer stage at baseline (NSCLC stage 1 or 2, NSCLC stage 3, NSCLC stage 4, or SCLC).

^2^Individuals had at most 30 days of risk of a hospital readmission after each hospital admission.

ICU, intensive care unit.

ED, emergency department.

CI, confidence interval.

### Palliative Care Referrals

**Figure 2:**
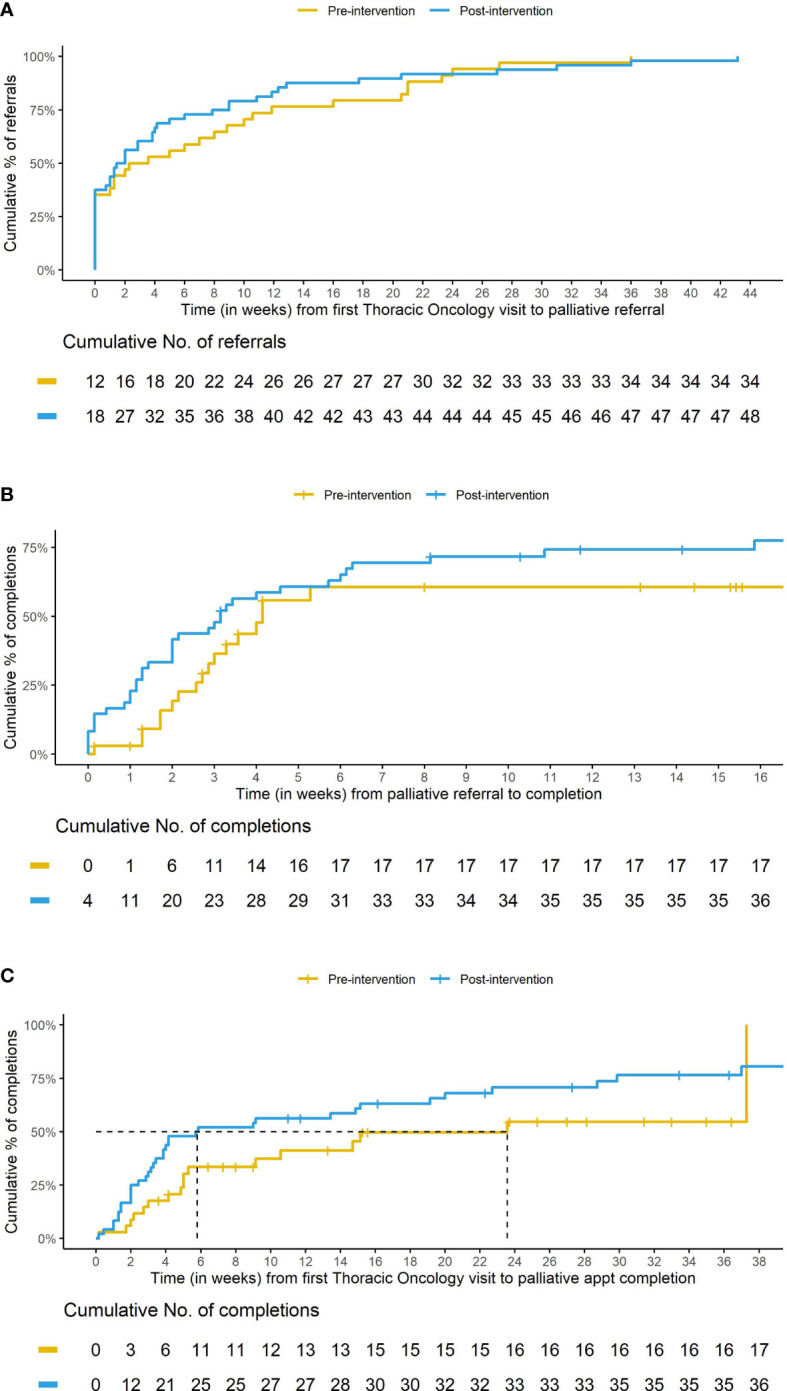
Cumulative incidence curves comparing pre- versus post-cohort. Caption: **(A)** Time (in weeks) from first Thoracic Oncology visit to palliative referral for patients with a palliative referral. Caption **(B)** Time (in weeks) from palliative referral to palliative appointment completion. Caption **(C)** Time (in weeks) from first Thoracic Oncology visit to palliative appointment completion for those with a palliative referral. Note the shorter length of time to median palliative appointment completion for those in the post- cohort. This decrease is likely due to patients being referred earlier **(A)** and completing their palliative appointment sooner **(B)**.

The proportion of palliative care referrals did not differ between the two cohorts, as 16% (34/214) of patients in the pre-cohort and 18% (48/260) of patients in the post-cohort had an ambulatory palliative care referral placed. Patients in the post-cohort were more likely to complete the ambulatory palliative care referral within the 1-year observation time period 75% (36/48) compared to the pre-cohort 50% (17/34). The median time from initial medical oncology appointment to placement of a palliative care referral was decreased in the post-cohort compared to the pre-cohort (12 days vs 21 days) (**Figure 2A**). Additionally, the median time from palliative care referral to palliative referral completion was also decreased in the post-cohort compared to the pre-cohort (22 days vs 29 days) (**Figure 2B**). With both earlier referrals and earlier palliative appointment completions following the referral, the median time from initial medical oncology appointment to palliative referral completion was substantially lower in the post-cohort compared to the pre-cohort (5.8 weeks vs 23.6 weeks) (**Figure 2C**).

To further evaluate the impact of embedded palliative care on ED visits, we analyzed emergency department visits before and after palliative care intervention in both cohorts (**Table 3**). This analysis included only those patients who completed a palliative care referral in the respective study period which consisted of 17 patients in the pre-cohort and 36 patients in the post-cohort. ED visits before palliative care consultation included any ED visit that occurred after the first Thoracic Oncology visit and prior to the patient being evaluated by palliative care and ED visits after palliative care consultation included any ED visit that occurred after a patient’s initial palliative care appointment and prior to the end of the study period. ED visits per-person-year decreased following palliative care intervention in both cohorts. ED visits decreased by approximately 50% in the pre-cohort (8.6 vs 4.1) and approximately 80% in the post-cohort (7.5 vs 1.5). ED visits following palliative care intervention decreased in the post-cohort, after the establishment of the embedded clinic model, compared to the pre-cohort, when only the stand-alone model existed (1.5 vs 4.1).

**Table 3:** Emergency department (ED) visits comparing pre- and post-cohort before and after palliative care consultation.

	Pre-cohort (n = 17)	Post-cohort (n = 36)
**Before palliative consultation**		
* Number of ED visits*	24	41
* Total observation time, years*	2.8	5.5
* ED visits per-person years*	8.6	7.5
**After palliative consultation**		
* Number of ED visits*	19	17
* Total observation time, years*	4.7	11.4
* ED visits per-person years*	4.1	1.5

ED, emergency department.

## Discussion

To our knowledge, this is the first study to evaluate the impact of a newly embedded onco-pall care model on healthcare utilization across the course of disease and not specifically focusing on end-of-life quality metrics. Early palliative care intervention is beneficial for patients with a new thoracic malignancy but there are still many challenges in care delivery. Prior research by Agne et al. identified “time burden to patients” as the primary logistical barrier to outpatient palliative care referral (18). In an effort to overcome this barrier, we established a palliative care clinic embedded within the thoracic medical oncology clinic. In contrast to prior studies (14), we found the embedded clinic model resulted in decreased healthcare utilization compared to the stand-alone clinic model.

We found a statistically significant decrease in ED visit rates in the post-cohort study period. This decrease may be due to improved access to palliative care and symptom management earlier in the disease course. Although only the outcome of ED visits was statistically significantly lowered, this study may not have been powered to detect a statistically significant difference in ICU and hospital admissions. However, using point estimates from the models, this translates into a 29% decrease in ICU admissions ($980k), 15% decrease in hospitalizations ($1.6 million), and a 26% decrease in ED visits ($86k) for a total of 2.7 million in annual savings. This observed decrease in healthcare utilization is both fiscally and clinically important. Additional research is warranted to further evaluate the fiscal impact of the embedded clinic model. Although ED visits, ICU visits, and hospital admissions were decreased, there was no observable impact on 30-day readmissions. One possible explanation for the lack of impact on 30-day readmissions is that patients are readmitted to the hospital shortly after discharge prior to being evaluated in the onco-pall clinic.

While there was no observed difference in the proportion of palliative care referral orders between the pre-cohort and the post-cohort, the frequency of completion of palliative care referral was increased in the post-cohort and the time from initial medical oncology appointment to completed palliative care appointment was substantially decreased in the post-cohort. Both the increased involvement of palliative care as well as the earlier involvement of palliative care could potentially contribute to the decrease in healthcare utilization outcomes observed in the post-cohort, as patients referred to palliative care had an outsized impact on healthcare utilization. For example, although 17% of all patients seen in the onco-pall clinic in the two years of the study were referred to palliative care, these patients accounted for 30% of all ED visits **(Appendix Table 2)**.

The involvement of multidisciplinary teams is recommended to provide high-quality cancer care and has been found to enhance communication among and improve knowledge among healthcare providers (19). Therefore, it is possible that the shared workspace for palliative care and medical oncology permitted additional informal input from palliative care which may have improved symptom control and contributed to the decrease in healthcare utilization even for those not formally seen by palliative care **(Appendix Table 2)**. For this reason, all patients seen in the onco-pall clinic in the respective time periods were included in the study rather than only those referred to palliative care. There is some evidence of this multidisciplinary team effect in our study in that patients seen in the pre-cohort who were never referred to palliative care had increased healthcare utilization compared to patients seen in the post-cohort who were never referred to palliative care.

Though it should be considered exploratory due to the small number of patients, data regarding the frequency of ED visits both before and after palliative care intervention provide additional support that the development of the embedded clinic model decreased rates of ED visits. When only the stand-alone clinic model was available, ED visits for patients seen by palliative care decreased by a factor of 2. After the development of the embedded clinic model, ED visits for patients seen by palliative care decreased by a factor of 5. While these results should be interpreted with caution due to the small sample size, patients referred to palliative care likely have advanced disease and greater symptom burden and therefore have a greater potential for high healthcare utilization. One possible explanation for the decrease in ED visits seen in the embedded clinic model is that this model may facilitate easier access to palliative care for those patients who may otherwise be too sick and less likely to complete palliative care or continue to seek care through the stand-alone model. Additionally, after establishing with palliative care, patients gain access to additional resources including palliative care pharmacists and palliative phone triage for additional symptom management.

Limitations of our study include the relatively small analytic sample size and inability to obtain healthcare utilization data that occurred at a site other than the academic medical center. The study also may not have been powered to detect differences in the healthcare utilization outcomes of hospital admissions and ICU admissions. Although this study would benefit from additional years of data, the impact of the COVID-19 pandemic would likely confound the results of this study. While we identified and controlled for several confounders, it is possible there were additional unmeasured confounders that affected these results. Additionally, reasons for palliative care referral were not collected as a part of this study and palliative care intervention in the embedded clinic model was provided by one palliative care physician. More details regarding reasons for palliative care referral and expansion of the embedded model to involve additional palliative care providers would be of benefit.

In conclusion, embedding palliative care within medical oncology has the potential to decrease healthcare utilization for patients with thoracic malignancies earlier in the disease course in addition to end-of-life outcomes.

## Data Availability Statement

The raw data supporting the conclusions of this article will be made available by the authors, without undue reservation.

## Ethics Statement

The studies involving human participants were reviewed and approved by The Ohio State University Institutional Review Board. Written informed consent for participation was not required for this study in accordance with the national legislation and the institutional requirements.

## Author Contributions

EB, JA, and CP: conceptualization. KG, JB, MG, SJ, MS, PK, EB, JA, and CP: data collection. JB and SJ: data analysis. KG and JB: drafting original version. EB, JA, and CP: supervision. All authors read and approved the final version of this manuscript.

## Funding

Funding for the development of the onco-pall clinic provided by private donor, Vicky Lippert. Research support was provided by the REDCap project and The Ohio State University Center for Clinical and Translational Science grant support (National Center for Advancing Translational Sciences, Grant UL1TR002733). CP is currently supported by the National Institute of Aging: 1K76AB074923-01. Research reported in this publication was supported by The Ohio State University Comprehensive Cancer Center and the National Institutes of Health under grant number P30 CA016058. We thank the Biostatistics Shared Resource (BSR) at The Ohio State University Comprehensive Cancer Center, Columbus, OH, for biostatistical support of this study. The funders were not involved in the study design, collection, analysis, interpretation of data, the writing of this article or the decision to submit it for publication.

## Conflict of Interest

The authors declare that the research was conducted in the absence of any commercial or financial relationships that could be construed as a potential conflict of interest.

## Publisher’s Note

All claims expressed in this article are solely those of the authors and do not necessarily represent those of their affiliated organizations, or those of the publisher, the editors and the reviewers. Any product that may be evaluated in this article, or claim that may be made by its manufacturer, is not guaranteed or endorsed by the publisher.
