# Supplementary material for: Impact of an Embedded Palliative Care Clinic on Healthcare Utilization for Patients With a New Thoracic Malignancy
Source: Front Oncol. 2022 Feb 28;12:835881. doi: 10.3389/fonc.2022.835881 (PMC8919515; doi:10.3389/fonc.2022.835881)
Supplement: Supplementary file 1 [file Table_1.docx]

**Appendix Table 1. Healthcare outcomes for patients residing within Franklin County only**

|  | **Number of events** | **Total person-years of exposure** | **Events per-person-year**  **(95% CI)** | **Relative risk**  **(95% CI)** | **Adjusted relative risk (95% CI)^1^** |
| --- | --- | --- | --- | --- | --- |
| **ICU admissions** |  |  |  |  |  |
| *Pre-cohort* | 27 | 51.6 | 0.52 (0.34-0.76) | Reference | Reference |
| *Post-cohort* | 21 | 60.0 | 0.35 (0.22-0.53) | 0.67 (0.35-1.27) | 0.73 (0.37-1.42) |
| **ED visits** |  |  |  |  |  |
| *Pre-cohort* | 194 | 51.6 | 3.76 (3.25-4.33) | Reference | Reference |
| *Post-cohort* | 172 | 60.0 | 2.87 (2.45-3.33) | 0.76 (0.57-1.02) | 0.76 (0.57-1.01) |
| **Hospital Admissions** |  |  |  |  |  |
| *Pre-cohort* | 239 | 51.6 | 4.63 (4.06-5.26) | Reference | Reference |
| *Post-cohort* | 228 | 60.0 | 3.80 (3.32-4.33) | 0.82 (0.65-1.04) | 0.82 (0.65-1.04) |
| **30-day readmissions^2^** |  |  |  |  |  |
| *Pre-cohort* | 50 | 7.4 | 6.74 (5.00-8.89) | Reference | Reference |
| *Post-cohort* | 46 | 6.6 | 7.00 (5.13-9.34) | 1.04 (0.67-1.60) | 1.12 (0.72-1.72) |

^1^ Adjusted for age, race (Non-Hispanic white vs other), marital status (married vs unmarried), sex (male vs female), location (patient primary address within Franklin County vs adjacent county), Charlson score at baseline, cancer type and cancer stage (NSCLC stage 1 or 2, NSCLC stage 3, NSCLC stage 4, or SCLC).

^2^ Individuals had at most 30 days of risk of a hospital readmission after each qualifying hospital admission.

ICU = intensive care unit.

ED = emergency department.

CI = confidence interval.
