# Supplementary material for: Impact of an Embedded Palliative Care Clinic on Healthcare Utilization for Patients With a New Thoracic Malignancy
Source: Front Oncol. 2022 Feb 28;12:835881. doi: 10.3389/fonc.2022.835881 (PMC8919515; doi:10.3389/fonc.2022.835881)
Supplement: Supplementary file 2 [file Table_2.docx]

**Appendix Table 2. Emergency department (ED) visits based on ambulatory palliative care status**

| **Palliative care status** | **Cohort** | **Number of patients** | **Number of ED visits** | **Total person-years of exposure** | **ED visits per-person-year** |
| --- | --- | --- | --- | --- | --- |
|  |  |  |  |  |  |
| **No referral** | Pre-intervention | 180 | 199 | 65.3 | 3.1 |
|  | Post-intervention | 212 | 163 | 75.0 | 2.2 |
|  |  |  |  |  |  |
| **Referral incomplete** | Pre-intervention | 17 | 28 | 6.0 | 4.6 |
|  | Post-intervention | 12 | 24 | 4.5 | 5.3 |
|  |  |  |  |  |  |
| **Referral completed** | Pre-intervention | 17 | 43 | 7.5 | 5.8 |
|  | Post-intervention | 36 | 58 | 16.9 | 3.4 |

ED=emergency department
